# Encephalopathy With Guillain-Barré Syndrome Secondary to Extensively Multidrug-Resistant Salmonella Typhi Infection: A Case Report

**DOI:** 10.7759/cureus.106203

**Published:** 2026-03-31

**Authors:** Ghadah A Al-Sharif, Mohamad Shieb, Fatima Mazahir, Jaidev Nath, Walid Abuhammour, Nidheesh Chencheri

**Affiliations:** 1 Pediatrics, Al Jalila Children's Speciality Hospital, Dubai, ARE; 2 Pediatric Infectious Diseases, Mayo Clinic, Rochester, USA; 3 Pediatric Infectious Diseases, Al Jalila Children's Speciality Hospital, Dubai, ARE; 4 Pediatrics Neurology, Al Jalila Children's Speciality Hospital, Dubai, ARE

**Keywords:** corticosteroids, encephalopathy, extensively drug-resistant, guillain-barré syndrome, intravenous immunoglobulin, pediatric infection, polyneuropathy, salmonella typhi, travel-related infection, typhoid fever

## Abstract

*Salmonella enterica* serotype Typhi (S. Typhi) primarily causes typhoid fever but can rarely lead to severe neurological complications. We report a 16-month-old boy who developed encephalopathy with Guillain-Barré Syndrome (GBS) following extensively drug-resistant (XDR) S. Typhi infection after travel to Pakistan. Initially presenting with diarrhea and low-grade fever, he was treated with oral cefixime, but deteriorated with high-grade fever. Blood cultures revealed XDR S. Typhi resistant to multiple antibiotics and sensitive only to meropenem and azithromycin. On admission, he became drowsy and experienced a seizure; lumbar puncture was unremarkable. High-dose dexamethasone was initiated for encephalopathy. He subsequently developed flaccid limb weakness and autonomic dysfunction, and nerve conduction studies confirmed axonal polyneuropathy consistent with GBS. Intravenous immunoglobulin (IVIG) was administered alongside supportive care, and a 21-day course of IV meropenem plus oral azithromycin was completed. The patient gradually regained motor function, and three months post-discharge, he was walking independently. This case illustrates a rare combination of encephalopathy and GBS due to XDR S. Typhi in a pediatric patient, highlighting the importance of early recognition, appropriate antimicrobial therapy, corticosteroids, and IVIG. Clinicians should remain vigilant for neurological complications in children from endemic regions, emphasizing timely diagnosis, tailored therapy, and preventive strategies.

## Introduction

*Salmonella enterica* serotype Typhi is a gram-negative bacterium first identified in 1829. The term "Typhi" derives from the Greek word typhus, meaning "smoky," a reference to the characteristic foggy mentation and delirium this pathogen can produce [1]. While this serotype is best known as the causative agent of typhoid fever, a systemic illness marked by gastrointestinal disturbance and nonspecific constitutional symptoms, it has long been recognized as a cause of serious neurological complications. These include bacterial meningitis, encephalitis, and peripheral neuritis [2].

The neurological burden of typhoid fever is compounded by the growing threat of antimicrobial resistance. Extensively drug-resistant (XDR) strains of S. Typhi have emerged as a significant concern, particularly following a well-documented outbreak in Pakistan in 2018, where susceptibility was largely restricted to azithromycin and carbapenems. The scope of this problem was already apparent before the outbreak; the World Health Organization reported that approximately 65% of S. Typhi isolates from symptomatic patients in Sindh province were extensively drug resistant. This narrowing of therapeutic options makes empiric management of severe or atypical typhoid presentations increasingly challenging [3]. Among the neurological complications of typhoid fever, which span meningitis, spastic weakness, seizures, and cerebellar dysfunction, acute polyradiculopathy stands out as exceptionally rare. The proposed pathophysiological mechanism is a non-T-cell-dependent process in which immunoglobulin (Ig)M antibodies directed against bacterial capsular components cross-react with myelin gangliosides, precipitating peripheral nerve injury [4].

## Case presentation

Our case involves a child who developed an extensively drug-resistant (XDR) *Salmonella Typhi* infection, initially presenting with gastroenteritis characterized by high-grade fever, vomiting, and diarrhea. The child was 16 months old, with an unremarkable medical history, no prior hospital admissions, and reassuring growth and development. He was born full term by a spontaneous normal vaginal delivery. The patient was initially treated at another healthcare facility as a standard case of typhoid gastroenteritis managed with symptomatic management, which included oral rehydration and antipyretics. However, on the fifth day of illness, the blood cultures confirmed XDR S. Typhi, prompting a switch to meropenem (40 mg/kg/dose every eight hours). Despite initiation of appropriate antibiotics, the patient’s condition rapidly deteriorated within a few hours, with the onset of altered sensorium and seizures, necessitating high-dose corticosteroid therapy (3 mg/kg loading dose, followed by 1 mg/kg every six hours for three days) on the same day.

**Figure 1:**
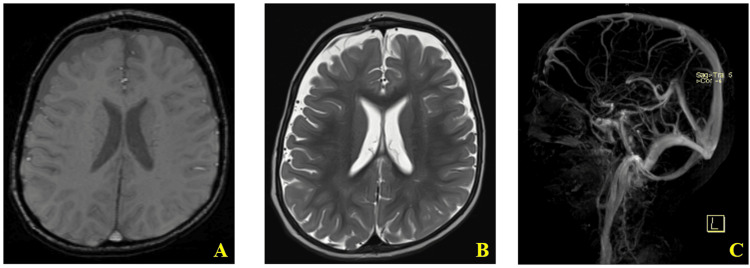
Brain MRI images of the patient at the presentation A. Normal brain MRI (axial plane, T2 flair).  B. Normal brain MRI with contrast (axial plane) showing the non-enhancing white matter. C. Normal brain magnetic resonance venography (MRV) with normal appearance of the major venous sinuses and superficial veins with no evidence of thrombosis, obstruction, filling defects, or infiltration.

In the context of systemic XDR S. Typhibacteremia, persistent altered mental status lasting more than 24 hours, and new-onset seizures, a diagnosis of Salmonella-associated encephalopathy was considered. Basic laboratory tests, including a complete blood count, kidney function, and electrolytes, were all within normal range. Cerebrospinal fluid (CSF) analysis and brain MRI (Figure 1) were unremarkable. Although these findings did not demonstrate definitive evidence of encephalitis, normal CSF and neuroimaging do not exclude central nervous system involvement, and the clinical presentation was most consistent with an acute inflammatory encephalopathic process.

Following a brain MRI, the patient developed autonomic dysfunction, including heart rate fluctuations and bradypnea with episodes of labored breathing during sleep, prompting transfer to the Pediatric Intensive Care Unit (PICU) one day later for close monitoring. During his PICU stay, he developed new-onset flaccid limb weakness with loss of deep tendon reflexes, which progressed over several days to axial hypotonia with marked head lag and inability to sit independently. Acute polyradiculopathy was suspected, and a lumbar puncture was performed, which revealed no abnormalities. Subsequently, electrodiagnostic evaluation, though technically challenging due to patient irritability, demonstrated markedly reduced compound action muscle potential (CMAP) amplitudes in the bilateral median nerves, inelicitable motor responses in the lower extremity nerves (peroneal and tibial), absent bilateral sural sensory nerve action potential (SNAP)s, and absent left median and ulnar sensory responses, consistent with a predominantly axonal polyneuropathy. A diagnosis of Guillain-Barré syndrome (GBS) was made, and intravenous immunoglobulin (IVIG) was initiated and received a total dose of 2 g/kg administered over two days, resulting in marked neurological improvement. This represents a rare case of XDR S. Typhi infection complicated by acute encephalopathy and Guillain-Barré syndrome, a combination scarcely described in the literature.

The child was also started on gabapentin for neuropathic pain. This approach was guided by the clinical presentation and supported by available evidence for managing immune‑mediated and neuropathic components of the condition [5]. He remained in the PICU for a total of four days, during which he did not require respiratory or inotropic support, and was subsequently transferred back to the general pediatrics ward. During his ward admission, the patient showed gradual improvement in motor strength. He received IV meropenem for 21 days and oral azithromycin for seven days, following recommendations from the pediatric infectious disease team.

The total hospital stay lasted 23 days, including four days in the PICU. At discharge, the patient’s motor power had returned to baseline; he was able to stand with support and sit from a lying position. According to his mother, he had regained his pre-illness functional status. He continued limb physiotherapy, and at his three-month follow-up, he demonstrated further improvement in movement transitions and was able to stand and walk independently.

## Discussion

Infections are strongly implicated as antecedent events in the development of Guillain-Barré syndrome (GBS), with up to two-thirds of cases reporting a symptomatic infection in the weeks preceding neurologic onset [6]. The most consistently identified trigger is *Campylobacter jejuni*, a bacterial cause of gastroenteritis linked to approximately 30-40% of GBS cases, and is thought to induce autoimmunity through molecular mimicry between bacterial lipooligosaccharides and peripheral nerve gangliosides [6]. Other bacterial and viral pathogens, including *Mycoplasma pneumoniae*, Cytomegalovirus, Epstein-Barr virus, Influenza virus, Hepatitis E virus, Zika virus, and more. Recently, Severe Acute Respiratory Syndrome Coronavirus 2 (SARS-CoV-2) has also been reported in association with GBS [7,8]. These infections are believed to provoke aberrant immune responses in susceptible individuals, leading to the production of cross-reactive antibodies that target components of peripheral nerves, resulting in demyelination and/or axonal injury [6]. Recent case reports and reviews describing GBS following SARS-CoV-2 infection further support the role of infection-associated immune dysregulation in the pathogenesis of GBS [8].

Panda PK investigated risk factors associated with the development of encephalopathy in children with S. Typhi infection [9]. The study identified several clinical and socioeconomic factors, including lower socioeconomic status, delayed initiation of treatment, malnutrition, elevated respiratory and heart rates, leukopenia, thrombocytopenia, a higher incidence of hepatic dysfunction, and elevated S. Typhi H and O agglutinin titers [9]. Notably, our patient shared several of these risk factors, including lower socioeconomic status, delayed initiation of appropriate therapy, and evidence of vital sign instability at presentation. Furthermore, Leung et al. reported that older children and young adults aged 10-25 years are at an increased risk of developing encephalopathy secondary to S. Typhi infection [10].

High-dose corticosteroids have been shown to reduce mortality in patients with severe typhoid fever [11]. The pathogenesis of severe typhoid involves dysregulated immune responses triggered by S. Typhi infection, leading to systemic inflammation and end-organ damage [12,13]. S. Typhi invades intestinal epithelial cells and persists within macrophages, evading innate immune defenses and disseminating systemically, with NF-κB signaling playing a key role in bacterial persistence [12]. Macrophages release pro-inflammatory cytokines, including interleukin-1β (IL-1β), interleukin-6 (IL-6), and tumor necrosis factor-alpha (TNF-α), contributing to systemic inflammation [12]. Excessive cytokine production can lead to sepsis and multi-organ dysfunction syndrome (MODS), which are commonly observed in severe typhoid cases [13]. High-dose corticosteroids, such as dexamethasone, exert potent anti-inflammatory effects by suppressing transcription of pro-inflammatory cytokines and inhibiting NF-κB activation, thereby modulating the host immune response and improving clinical outcomes [11]. Additionally, venous congestion and cerebral edema, frequent complications of S. Typhi encephalopathy, have been shown to respond favorably to corticosteroid therapy [11].

Furthermore, dexamethasone has demonstrated benefit in severe typhoid fever, independent of encephalopathy. A randomized trial conducted in Jakarta among patients with typhoid fever and sepsis showed that high-dose dexamethasone significantly reduced mortality compared with placebo [12]. Although high-dose dexamethasone is not routinely recommended for acute respiratory distress syndrome (ARDS) associated with typhoid fever, a case report of a previously healthy 16-year-old patient with typhoid-associated septic shock and ARDS documented marked clinical improvement following initiation of a high-dose dexamethasone regimen [14]. It is important to note that while low-dose dexamethasone has been proposed as an alternative, a retrospective review found no survival benefit in patients with severe typhoid fever [12].

The rationale for using intravenous immunoglobulin (IVIG) in post-infectious Guillain-Barré Syndrome (GBS) and related conditions, such as Bickerstaff’s brainstem encephalitis, centers on its ability to modulate the immune system by delivering concentrated donor antibodies that can physically block harmful autoantibodies from targeting neural cells or interfere with inflammatory pathways [15,16]. In GBS, the sources demonstrate that IVIG specifically protects sympathetic neurons from antibody-mediated overactivity by blocking the upregulation of tyrosine hydroxylase, the rate-limiting enzyme for noradrenaline synthesis, thereby neutralizing potential autonomic dysfunction [17]. While S. Typhi and Paratyphi A are established triggers for these autoimmune neurological syndromes, evidence from a case involving *Salmonella dublin* confirms that IVIG is a central immunotherapy for recovery; a patient presenting with flaccid paralysis and impaired consciousness showed significant neurological improvement after receiving two courses of IVIG [15]. This treatment facilitated the gradual return of motor function and speech, eventually allowing the patient to walk independently, which underscores the efficacy of IVIG in treating rare, invasive Salmonella-associated neurological complications [15].

The manifestation of GBS post S. Typhi infection was first discussed in 1969 in an adult patient [16]. The literature reports only a very limited number of cases of Guillain‑Barré syndrome (GBS) as a rare complication of typhoid fever in children. Mehndiratta et al. [4] and Kapoor et al. [17] described cases in which children with S. Typhi enteritis confirmed by blood culture developed GBS. Mehndiratta et al. reported a 30-month-old patient whose neurological examination was largely normal except for reduced motor power in the lower limbs bilaterally [4]. In contrast, the 18-month-old patient described by Kapoor et al. exhibited more severe neurological deficits, including flaccid quadriplegia with a motor power of 0/5 [17]. Both cases demonstrated elevated cerebrospinal fluid protein and electrodiagnostic findings consistent with GBS, showing a demyelinating pattern affecting motor and sensory nerves. Initial management with antibiotics alone did not result in improvement; however, both patients showed dramatic clinical and neurological recovery following treatment with intravenous immunoglobulin (IVIG), regaining neurological power after therapy. In our patient, the decision to initiate IVIG was guided by the presence of radiculopathy manifestations consistent with acute inflammatory demyelinating polyradiculopathy, including sudden proximal and truncal muscle weakness [4,16,17].

Regarding prevention, typhoid conjugate vaccines (TCVs) are now recommended in endemic regions and can be administered starting at six months of age, offering broader protection in young children. However, vaccine availability, implementation policies, and access may vary across regions. In addition to vaccination, preventive strategies continue to rely on safe food and water practices and strict hygiene measures, particularly for young children traveling to endemic areas [18].

## Conclusions

The occurrence of both encephalitis and Guillain‑Barré Syndrome as complications of typhoid fever is exceedingly rare. The patient’s recent travel to Pakistan, a region with a high prevalence of extensively drug-resistant Salmonella Typhi, highlights the importance of heightened vigilance among healthcare providers in diagnosing and managing such complex cases. The initial misdiagnosis and subsequent treatment with inadequate cefixime may have contributed to the progression to encephalitis and polyneuropathy. High-dose dexamethasone and IVIG proved to be critical components of the patient’s therapeutic regimen. Although neurological complications of typhoid fever are uncommon, high-dose dexamethasone has shown potential in reducing mortality and alleviating typhoid-induced encephalopathy. IVIG, while not typically indicated for Salmonella encephalopathy, was administered in this case due to the presence of polyneuropathy consistent with Guillain‑Barré Syndrome.

## References

[1] Ashurst JV, Truong J, Woodbury B (2023). Typhoid Fever (Salmonella Typhi) (Archived). https://pubmed.ncbi.nlm.nih.gov/30085544/.

[2] Ali G, Rashid S, Kamli MA, Shah PA, Allaqaband GQ (1997). Spectrum of neuropsychiatric complications in 791 cases of typhoid fever. Trop Med Int Health.

[3] (2026). Typhoid fever - Pakistan. World Health Organization.

[4] Mehndiratta S, Rajeshwari K, Dubey AP (2012). Guillain-Barré syndrome as a complication of typhoid fever in a child. Neurol India.

[5] Fleser L, Tibbetts E, Hanson A (2024). Evaluating gabapentin dosing, efficacy and safety in infants. J Pediatr Pharmacol Ther.

[6] Willison HJ, Jacobs BC, van Doorn PA (2016). Guillain-Barré syndrome. Lancet.

[7] van den Berg B, Walgaard C, Drenthen J, Fokke C, Jacobs BC, van Doorn PA (2014). Guillain-Barré syndrome: pathogenesis, diagnosis, treatment and prognosis. Nat Rev Neurol.

[8] Censi S, Bisaccia G, Gallina S, Tomassini V, Uncini A (2024). Guillain-Barré syndrome and SARS-CoV-2 infection: a systematic review and meta-analysis on a debated issue and evidence for the 'Italian factor'. Eur J Neurol.

[9] Panda PK, Panda K (2018). Study of clinico‑epidemiological risk factors associated with enteric encephalopathy in children. Int J Contemp Ped.

[10] Leung DT, Bogetz J, Itoh M, Ganapathi L, Pietroni MA, Ryan ET, Chisti MJ (2012). Factors associated with encephalopathy in patients with Salmonella enterica serotype Typhi bacteremia presenting to a diarrheal hospital in Dhaka, Bangladesh. Am J Trop Med Hyg.

[11] Nain Z (2023). High‑dose dexamethasone in complicated typhoid fever: what is the evidence?. Indian J Pediatr.

[12] Bossel Ben-Moshe N, Hen-Avivi S, Levy Efrati L (2025). Salmonella Typhi gut invasion drives hypoxic immune subsets associated with disease outcomes. Nat Commun.

[13] Stepien TA, Singletary LA, Guerra FE (2024). Nuclear factor kappa B-dependent persistence of Salmonella Typhi and Paratyphi in human macrophages. mBio.

[14] Ugas MB, Carroll T, Kovar L, Chavez-Bueno S (2016). Salmonella Typhi-induced septic shock and acute respiratory distress syndrome in a previously healthy teenage patient treated with high‑dose dexamethasone. J Investig Med High Impact Case Rep.

[15] Xie J, Zhang T, Liu T (2021). First report of Bickerstaff's brainstem encephalitis caused by Salmonella Dublin: a case report. BMC Neurol.

[16] Chanmugam D, Waniganetti A (1969). Guillain-Barré syndrome associated with typhoid fever. Br Med J.

[17] Kapoor K, Jain S, Jajoo M, Talukdar B (2014). A rare neurological complication of typhoid fever: Guillain-Barre' syndrome. J Pediatr Neurosci.

[18] (2026). Immunization, Vaccines and Biologicals. https://www.who.int/teams/immunization-vaccines-and-biologicals/diseases/typhoid?utm_source=chatgpt.com.

